# Case Report: Spontaneous colonic rupture in a 12-year-old competitive swimmer with vascular Ehlers–Danlos syndrome

**DOI:** 10.3389/fped.2026.1883212

**Published:** 2026-10-13

**Authors:** Keke Run, Yi Yuan, Xianli Wen, Xueyian Hu, Danrong Zhu

**Affiliations:** Children’s Medical Center, The Second Affiliated Hospital of Nanjing Medical University, Nanjing, China

**Keywords:** case report, *COL3A1*, Hartmann’s procedure, pediatrics, spontaneous colonic rupture, vascular Ehlers–Danlos syndrome

## Abstract

**Introduction:**

Vascular Ehlers–Danlos syndrome (vEDS) is a rare connective tissue disorder caused by *COL3A1* pathogenic variants, characterized by pronounced fragility of arteries, intestines, and uterus. Spontaneous colonic rupture in pediatric patients is extremely rare and easily misdiagnosed. Definitive diagnosis remains challenging in patients with nonspecific phenotypes. Molecular genetic testing is essential for diagnostic confirmation and may inform genotype-associated risk assessment.

**Case presentation:**

We present a 12-year-old competitive swimmer with sudden lower abdominal pain following intensive training. Initial non-contrast CT showed bowel wall thickening and mild intraperitoneal fluid without overt perforation, leading to conservative management. Despite subjective pain improvement, her hemoglobin level declined markedly on the second hospital day. Contrast-enhanced CT showed increased free fluid and progressive bowel lesions. Emergency laparotomy revealed spontaneous rupture at the descending–sigmoid colon junction with hemoperitoneum. Given the severe intra-abdominal contamination, active hemorrhage, and unfavorable local conditions for safe primary anastomosis, Hartmann's procedure was performed. The subsequent diagnosis of vEDS further supported the appropriateness of avoiding primary anastomosis in this patient. Genetic testing identified a heterozygous pathogenic *COL3A1* variant, confirming vEDS.

**Conclusion:**

This case emphasizes that transient subjective improvement may mask progressive gastrointestinal injury in children with vEDS. Serial hemoglobin decline, progressive imaging abnormalities, and evolving hemodynamic instability should prompt urgent surgical assessment.

## Introduction

Vascular Ehlers–Danlos syndrome (vEDS) is a rare, life-threatening connective tissue disorder caused by pathogenic variants in the *COL3A1* gene, characterized by arterial, intestinal, and uterine fragility. The estimated prevalence is approximately 1 in 50,000 to 1 in 200,000 (1–3), with most patients presenting in adolescence or early adulthood. Spontaneous colonic rupture in childhood is exceedingly rare, especially in the absence of antecedent trauma or surgical history.

Here, we describe a 12-year-old competitive swimmer who presented with acute abdominal pain and was ultimately found to have spontaneous colonic rupture due to vEDS. The patient's athletic background, transient symptom improvement, and subtle abdominal signs initially obscured the severity of her condition, delaying recognition. This case underscores the importance of including vascular fragility disorders in the differential diagnosis of acute abdomen in children, even when the initial examination and routine coagulation profile are unremarkable.

## Case presentation

A 12-year-old competitive swimmer developed sudden lower abdominal pain following intensive training. Initial non-contrast computed tomography (CT) revealed an indeterminate mass lesion in the descending colon alongside pelvic effusion, with no definitive radiological evidence of perforation; conservative management was therefore initiated. Nevertheless, the child still suffered from severe intolerable abdominal pain in the early hours of the next day. The persistent pain was localized to the left lower quadrant and progressed gradually. The patient was forced to adopt a curled posture and became unable to ambulate, accompanied by cessation of defecation and flatus, which prompted hospital admission (Figure 1A). On admission, her temperature was 37.3° C, heart rate was 110 beats/min, respiratory rate was 23 breaths/min and oxygen saturation was 99% on room air. She was alert. Abdominal examination showed no abdominal distension, with tenderness localized to the left lower quadrant and lower abdomen. Rebound tenderness and involuntary guarding were absent. Bowel sounds were hypoactive. No cutaneous stigmata and characteristic facial features suggestive of vEDS were identified on initial examination. Six months prior to admission, the patient reportedly underwent thrombectomy for axillary arterial thrombosis and remained on oral anticoagulation thereafter.

**Figure 1:**
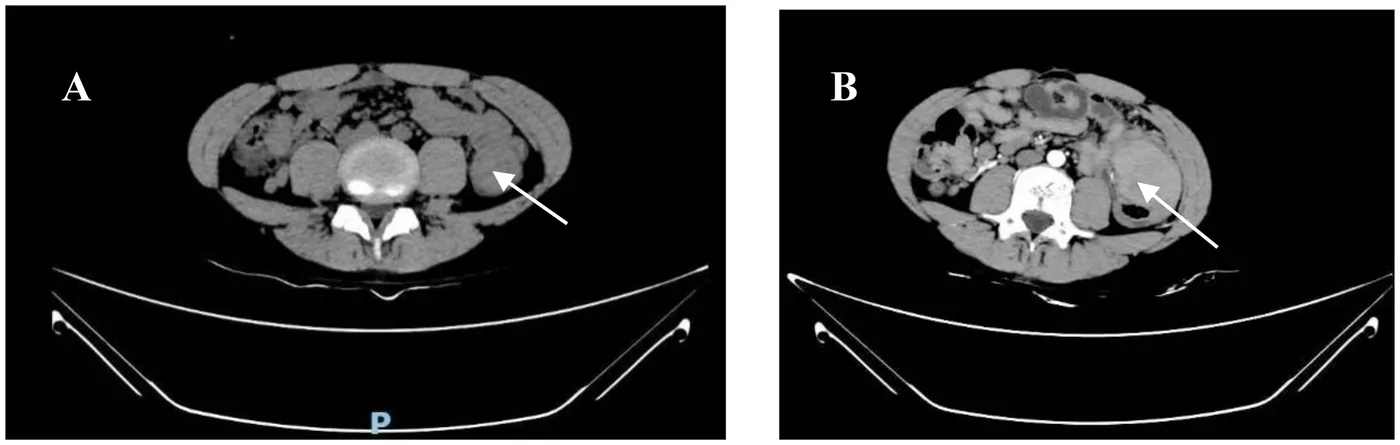
**(A)** Initial non-contrast CT showed an indeterminate mass-like lesion in the descending colon. **(B)** Contrast-enhanced CT showed increased free intraperitoneal fluid, and the mass-like lesion in the descending colon was markedly enlarged compared with that on the initial CT.

On the second hospital day, although her abdominal pain improved subjectively, she continued to have neither defecation nor passage of flatus. Her hemoglobin level decreased from 122 to 91 g/L. These findings raised strong clinical suspicion of ongoing intra-abdominal hemorrhage; therefore, emergency contrast-enhanced abdominal CT was performed. Contrast-enhanced CT showed increased free intraperitoneal fluid and worsening bowel wall abnormalities (Figure 1B). After urgent preoperative assessment, emergency exploratory laparotomy was performed. Massive intraperitoneal hemorrhage and numerous blood clots were observed. Careful exploration directly identified an approximately 8 cm full-thickness rupture at the descending–sigmoid colon junction, which was covered by extensive blood clots (Figure 2). Hartmann's procedure was performed because of the colonic rupture, severe intra-abdominal contamination, active hemorrhage, and unfavorable local conditions for primary anastomosis. A pelvic drain and a left paracolic gutter drain were placed, and the stoma was temporarily dressed with oil-impregnated gauze. Histopathologic analysis demonstrated chronic inflammatory changes confined to the mucosa. Diffuse and extensive hemorrhage was observed throughout the full thickness of the intestinal wall as well as within the intestinal lumen, with concomitant hematoma formation (Figure 3). Postoperatively, routine stoma and pouch care was provided. Six months later, the patient underwent colostomy reversal and recovered from the acute surgical episode. Postoperative genetic testing identified a heterozygous pathogenic variant in *COL3A1* (*c.2553* *+* *1G > C*), confirming the diagnosis of vascular Ehlers–Danlos syndrome (vEDS). Targeted testing of the patient's younger brother 1 month later did not detect the familial *c.2553* *+* *1G > C* variant, while the patient's parents refused consent for any relevant genetic testing owing to personal concerns of the family. No relevant familial medical history was reported by either parent.

**Figure 2:**
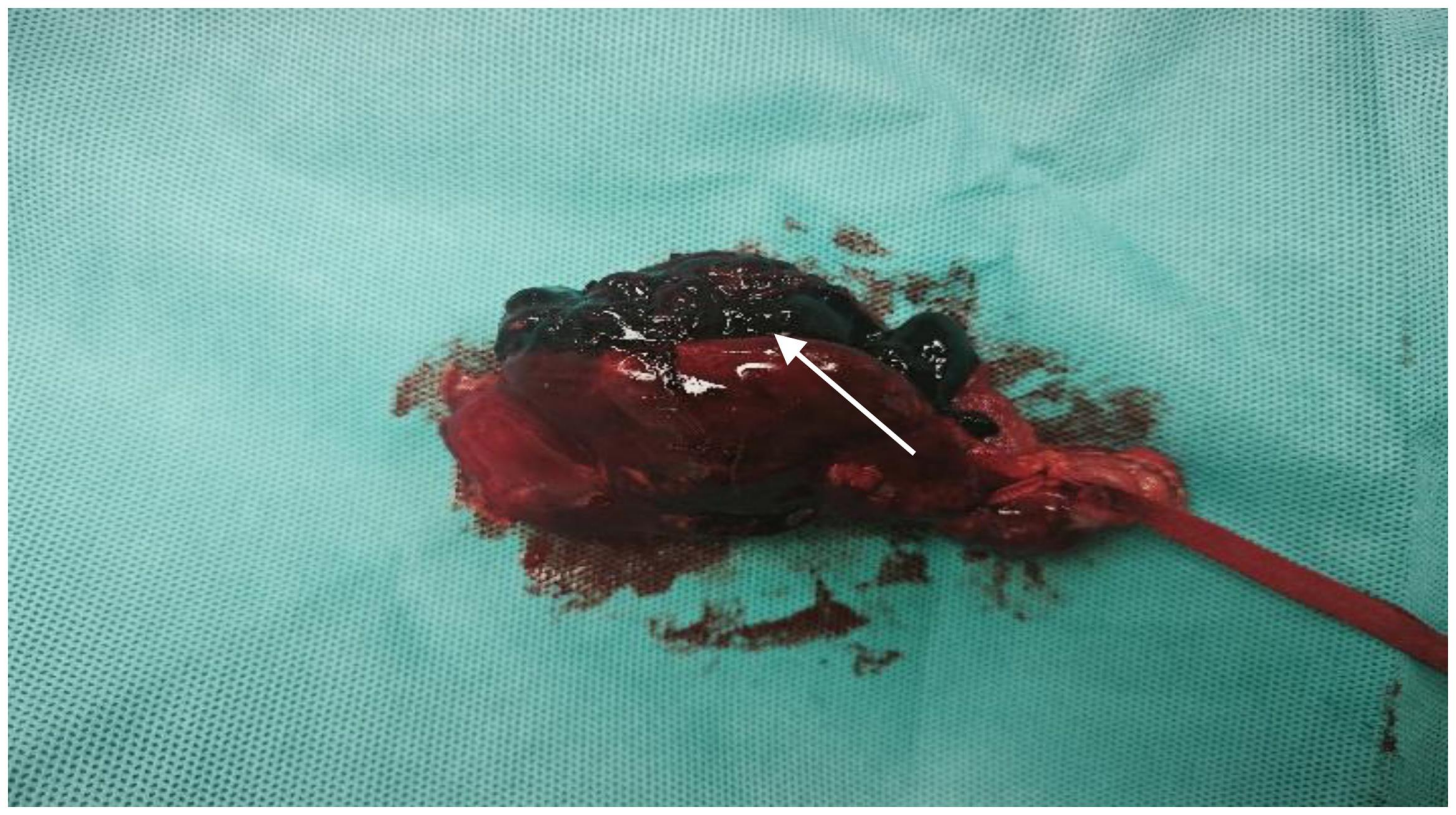
Resected surgical specimen showing a longitudinal rupture of the sigmoid colon.

**Figure 3:**
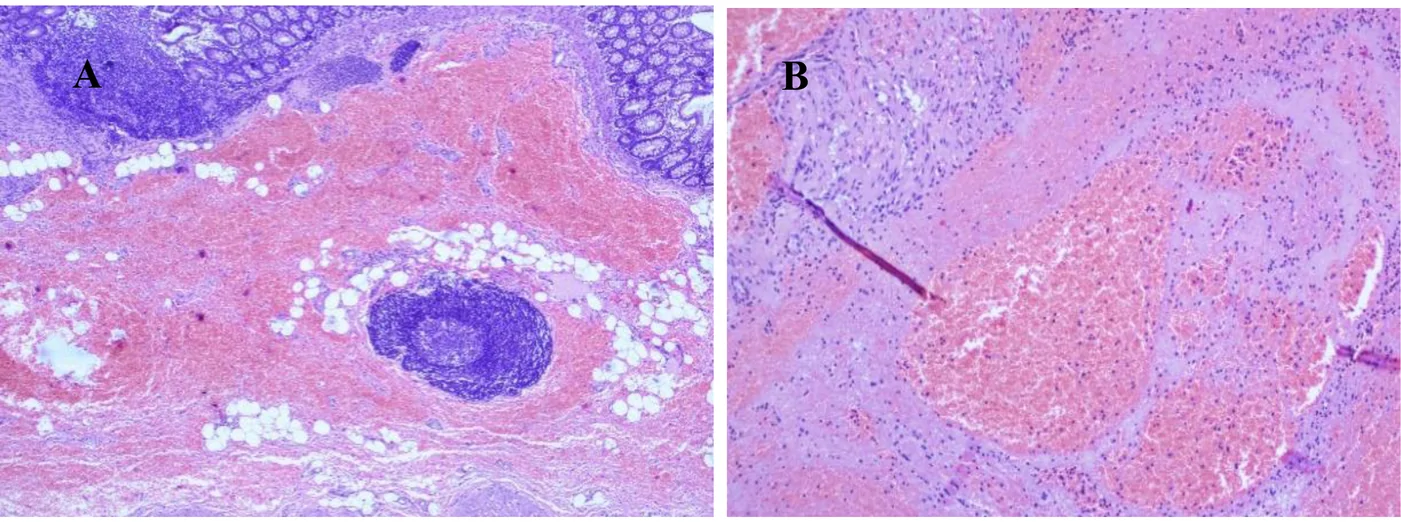
**(A)** Observation under 40-fold magnification shows transmural hemorrhage throughout the intestinal wall. **(B)** Observation under 100-fold magnification shows transmural hemorrhage throughout the intestinal wall.

Following genetic confirmation of vEDS, the patient and her family received genetic counseling. She discontinued competitive swimming and was advised to avoid high-intensity, collision, and heavy-resistance activities because of the risk of vascular or visceral injury. Long-term multidisciplinary follow-up involving clinical genetics, vascular medicine or surgery, pediatric surgery, and gastroenterology was recommended. The clinical course is summarized in Figure 4.

**Figure 4:**
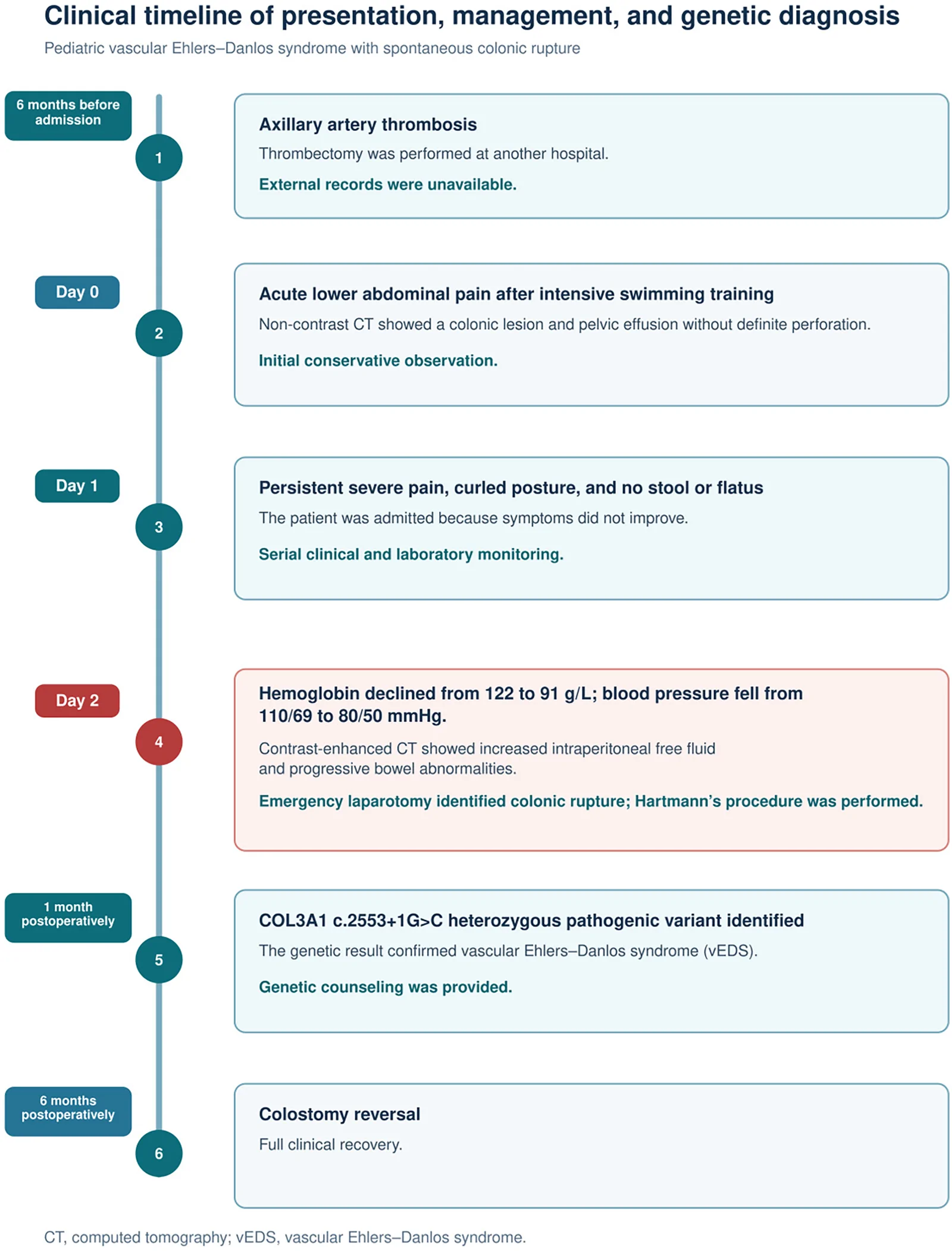
Clinical timeline of presentation, management, and genetic diagnosis in a pediatric patient with vascular Ehlers–Danlos syndrome and spontaneous colonic rupture. CT, computed tomography; vEDS, vascular Ehlers–Danlos syndrome.

## Discussion

Ehlers–Danlos syndromes (EDS) are a heterogeneous group of connective tissue disorders caused by genetic defects affecting collagen structure and extracellular matrix integrity. Currently, 13 clinical subtypes and 19 pathogenic genes have been defined (1, 4). Among them, vascular EDS (type IV) represents the most severe subtype, with an estimated prevalence of approximately 1 in 50,000 to 1 in 200,000, accounting for less than 4% of all EDS cases. vEDS is caused by *COL3A1* mutations inherited in an autosomal dominant pattern, with approximately 50% of cases resulting from *de novo* variants (2, 3). Classic clinical features of vEDS include thin translucent skin, easy bruising, characteristic facial features, arterial dissection or rupture, gastrointestinal perforation, pneumothorax, and early-onset varicose veins. Notably, skin and joint manifestations are often mild or absent, contributing to frequent underdiagnosis, especially in children (1–3, 5). This pediatric patient did not exhibit the characteristic facial features of vEDS, such as thin lips, a narrow nose, prominent eyes, or micrognathia, and presented only with a lifelong history of easy bruising; a definite diagnosis of vEDS had not been established before this acute event.

Our patient developed two life-threatening events within 6 months: axillary artery thrombosis and spontaneous colonic rupture. She also had a history of easy bruising and skin abrasions during childhood. In retrospect, the prior axillary arterial event raised suspicion of underlying vascular fragility associated with vEDS. However, the detailed imaging findings, operative records, pathological data, and thrombophilia evaluation from the previous hospitalization were unavailable. Therefore, the exact mechanism of the axillary thrombosis could not be determined. Given the patient's history of competitive swimming, repetitive upper-extremity exertion and arterial thoracic outlet syndrome should also be considered as alternative or contributory explanations. The temporal relationship between intensive training and the current abdominal event similarly cannot establish causality. In symptomatic vEDS patients, arterial complications are the most common (63%), followed by gastrointestinal involvement (26%) (6). Approximately 85% of gastrointestinal complications present as spontaneous colonic perforation, which predominantly affects the sigmoid colon. Although intestinal perforation is rare in early childhood, its incidence increases in older children and young adults, affecting 25%–30% of patients over their lifetime. Spontaneous colonic perforation typically first manifests in late teens to the third decade of life. The majority of patients are genetically confirmed to have vEDS subsequent to perforation onset, as seen in the present case. Variable disease severity is observed among different *COL3A1* mutations, with a small subset of patients experiencing perforation at middle or advanced age. In the presence of confirmed perforation, active hemorrhage, or hemodynamic deterioration, timely surgical intervention may be lifesaving (3). Despite the necessity of emergent surgery, patients face marked perioperative risks driven by tissue fragility and defective wound repair, with a re-perforation rate of up to 55% following limited segmental resection (7). No standardized surgical paradigm has been established for this clinical entity. A systematic review by Speake et al. suggested that total abdominal colectomy with end ileostomy or ileorectal anastomosis may reduce the risk of recurrent colonic perforation, whereas procedures leaving the remaining colon *in situ* may carry a substantial risk of reperforation. However, the evidence is derived mainly from retrospective reports and small case series, and the operative strategy should therefore be individualized (8). Therefore, although more extensive colonic resection may reduce the risk of recurrent colonic perforation in selected patients, routine prophylactic completion colectomy after a single event cannot be universally recommended on the basis of the currently limited evidence. For this patient, any future surgical intervention should be considered only after individualized multidisciplinary assessment, taking into account recurrent symptoms, the extent of residual colonic disease, vascular risk, and the substantial operative risks associated with tissue fragility.

One case reported by Yoshizaki and colleagues described a genetically verified vEDS male complicated with sigmoid perforation-triggered ruptured dissecting splenic artery aneurysm; the patient survived after emergency Hartmann operation (9). At the time of emergency surgery, vEDS had not yet been diagnosed. Hartmann's procedure was selected because of severe intra-abdominal contamination, active hemorrhage, and unfavorable local conditions for safe primary anastomosis; the subsequent diagnosis of vEDS further supported the decision to avoid primary anastomosis. In vEDS-associated colonic perforation, operative management should be individualized according to hemodynamic status, extent of colonic involvement, contamination, tissue quality, and the risk of recurrent perforation. Available approaches include Hartmann's procedure, segmental colectomy with diversion, primary repair with a diverting stoma, and total colectomy with end ileostomy in selected patients. This cohort carries a high risk of postoperative complications such as intraperitoneal bleeding, fascial dehiscence, enterocutaneous fistulas, and peritoneal adhesions (10). Although restoration of intestinal continuity may carry risks of anastomotic complications and recurrent perforation in vEDS, stoma reversal was undertaken 6 months later after individualized multidisciplinary assessment. The patient and family were counseled regarding these risks, and close long-term follow-up was arranged.

Hemorrhagic complications in vEDS may initially be occult and may precede overt peritoneal signs. Accordingly, serial hemoglobin measurement and hemodynamic monitoring should be considered in patients with suspected vEDS who present with unexplained abdominal pain, particularly when symptoms and objective findings are discordant (3, 7).

The potential influence of previous oral anticoagulation on the severity of hemorrhage could not be reliably determined because the specific agent, dose, and timing of the last dose were unavailable. Normal routine coagulation tests did not definitively exclude a residual anticoagulant effect. Nevertheless, anticoagulation might have aggravated the severity of bleeding but would not account for the structural colonic rupture.

The *COL3A1* gene encodes the pro-α1 chains of type III collagen, a key structural component in blood vessels, gastrointestinal tract, uterus, and other hollow organs. *COL3A1* mutations lead to abnormal synthesis, structure, or secretion of type III collagen, resulting in weakened vessel and visceral walls and increased susceptibility to spontaneous rupture (6, 7, 11, 12). The type of *COL3A1* variant influences the disease course and clinical phenotype of vascular Ehlers–Danlos syndrome (vEDS). Heterozygous missense mutations exhibit a higher severity of pathogenicity compared to splicing mutations and haploinsufficiency mutations, resulting in an earlier age at initial diagnosis. This may be attributed to the trimeric molecular structure of type III collagen encoded by *COL3A1*. Heterozygous missense mutations primarily involve substitutions of glycine residues. When glycine is replaced by larger charged amino acids, such as aspartic acid, the triple-helical structure of collagen is severely disrupted, leading to the highest disease severity (6, 13, 14).

The patient in this study carried a splice-site variant c.*2553* *+* *1G > C* in *COL3A1*. Approximately one quarter of reported pathogenic *COL3A1* variants occur at splice sites, and most result in exon skipping. Such variants may disrupt type III collagen assembly and can be associated with severe clinical phenotypes. The *c.2553* *+* *1G > C* variant was absent from large-scale population databases and was classified as pathogenic in the patient's genetic report (6, 13, 14).

Vascular Ehlers–Danlos syndrome (vEDS) is a rare disorder with no curative treatment currently available. Clinical manifestations are highly variable and multisystemic, leading to misdiagnosis and delayed diagnosis, especially in children with nonspecific phenotypes. Molecular genetic testing is essential for confirming the diagnosis of vEDS and may assist in genotype-associated risk assessment. The *c.2553* *+* *1G > C* variant was absent from large-scale population databases, supporting its rarity; its pathogenic classification was based on the molecular genetic report and the patient's compatible clinical phenotype (3, 6, 12, 13).

Following recovery from the acute surgical episode and subsequent stoma reversal, this patient remains at lifelong risk of potentially fatal complications, including arterial dissection or rupture, recurrent gastrointestinal perforation, and pregnancy-associated uterine or vascular rupture. A large retrospective cohort reported a median survival of approximately 50 years in patients with vEDS; however, survival varies substantially according to the type and molecular consequence of the COL3A1 variant, with considerable interindividual and intrafamilial variability (13). Therefore, this population-level estimate cannot be used to predict the life expectancy of this individual patient, and her long-term outcome remains uncertain.

Long-term management should emphasize coordinated multidisciplinary follow-up, regular blood-pressure monitoring, individualized noninvasive arterial surveillance, genetic counseling, and prompt assessment of sudden unexplained chest, abdominal, back, head, or limb pain (3, 15). Patients should avoid collision sports, competitive high-intensity activities, and heavy resistance exercise. No established prophylactic endoscopic intervention has been shown to prevent recurrent colonic rupture. Routine colonoscopy should generally be avoided in the absence of a compelling clinical indication because of the risk of iatrogenic perforation (3). Similarly, elective surgery should be considered only when the anticipated benefit clearly outweighs the substantial risks related to vascular and tissue fragility. Because vEDS is inherited in an autosomal dominant manner, targeted genetic testing and counseling should be offered to first-degree relatives. In the present family, the patient's younger brother tested negative for the familial variant, whereas parental testing was declined; consequently, the variant could not be classified as inherited or *de novo*.

In conclusion, this case demonstrates that transient symptomatic improvement may mask progressive gastrointestinal injury in pediatric vEDS. Serial hemoglobin decline, progressive imaging abnormalities, and evolving hemodynamic instability should prompt urgent surgical assessment. Early recognition, genetic confirmation, and multidisciplinary management are essential to improve outcomes in children with vEDS.

## Data Availability

The datasets presented in this study can be found in online repositories. The names of the repository/repositories and accession number(s) can be found in the article.
